# Continuous hypoxia reduces the concentration of streptomycin in the blood

**DOI:** 10.1186/s12879-018-3027-7

**Published:** 2018-03-09

**Authors:** Lin Chen, Zhancheng Gao

**Affiliations:** 1 Department of Respiratory and Critical Care Medicine, Sichuan Academy of Medical Sciences & Sichuan Provincial People’s Hospital, Chengdu, 610072 China; 2 Department of Respiratory and Critical Care Medicine, Peking University People’s Hospital, 11 Xi Zhi Men Nan Da Jie, Beijing, 100044 China

**Keywords:** Streptomycin, Hypoxia, Pharmacokinetics, Plateau areas

## Abstract

**Background:**

A high incidence and mortality of plague in the past two decades occurred in the Qinghai-Tibet Plateau, China. High dose streptomycin (6–8 g/d) remained the first practical strategy for controlling the progressive, vicious clinical circumstances for patients with pneumonic plague in the Plateau, as opposed to the routine dosage recommended by the World Health Organization. To investigate whether patients with pneumonic plague truly required a large dosage of streptomycin in the hypoxic environment of the Tibetan Plateau, we investigated the hypothesis that hypoxic environment would change the pharmacokinetics of streptomycin in vivo.

**Methods:**

(1) We retrospectively analyzed the data of pneumonic plague patients administered streptomycin from January 1, 2000 to December 31, 2012 in these areas, which came from the database of the Qinghai Center for Disease Control; and (2) We used a persistent hypoxia chamber to simulate the plateau hypoxic environment and fed Sprague Dawley rats in the chambers for one month. Then, we continuously administered hypoxic rats a single loading dose (200 mg/kg) of streptomycin and analyzed its concentrations by high performance liquid chromatography. The pharmacokinetic profiles were analyzed using a non-compartmental method in the Phoenix WinNonlin program.

**Results:**

(1) There were 32 cases of patients with pneumonic plague in the past two decades totally and 9 of them died (all-cause mortality 28.125%, 9/32), including 7 cases died of delayed diagnosis without treatment of streptomycin, and the only 2 patients received normal dose of streptomycin. (2) The pharmacokinetic behaviors of streptomycin were different between the hypoxic and normal rats. Administration in a hypoxic state resulted in 74.81% and 29.28% decreases in maximum plasma concentration and area under the concentration-time curve from time zero to infinity compared with those values under normal condition for streptomycin.

**Conclusions:**

These results indicated that hypoxic condition could significantly decrease the absorption rate and extent of streptomycin. Therefore, patients with pneumonic plague require higher doses of streptomycin to maintain effective drug concentrations in Qing Hai and the Tibetan Plateau.

## Background

The mean altitude of the Qinghai-Tibet Plateau is 4000–5000 m. This area is one of the most active foci of plague. Pneumonic plague is the most common type of plague in this area. In China, the morbidity from pneumonic plague is 57.37%. Patients with pneumonic plague die within two or three days unless treated immediately.

Streptomycin has been used to treat plague for more than 60 years. In 1999, the World Health Organization (WHO) has recommended streptomycin as the most preferred and effective drug for various types of plague [1] .Since then, the mortality of plague has been reduced from 50 to 90% to less than 5% [2] .For the treatment of pneumonic plague, the dose of streptomycin in the Plateau region is problematic. The dose of streptomycin recommended by WHO is 2 g/d [1, 3] .However, the effective dose of streptomycin used in the Plateau region of China is 6–8 g/d, without serious side effects [4, 5] .Conversely, it may delay recovery in the Qinghai-Tibet Plateau to administrate normal dose of streptomycin for treating pneumonic plague, and/or leading to septic shock and multiple organ failure. Therefore, the dose of streptomycin recommended by the Chinese Center for Disease Control and Prevention (CDC) is much higher than that by the WHO [4]

.It arises from local physicians’ clinical experiments to treat pneumonic plague with high-dose of streptomycin. Many case studies have shown that high-dose of streptomycin could effectively control the disease without many side effects. Therefore, the CDC recommended high dosage streptomycin in 2009, but more evidence was needed to confirm its effectiveness.

As the above, normal dose of streptomycin is effective enough to control pneumonic plague in the plains, but not in the plateau. And as we know, it is a hypoxia environment in the plateau, which is much different from the plains. Then, we hypothesized that the hypoxia environment might influence on the effectiveness of streptomycin. Therefore, we firstly retrospectively analyzed the clinical data of pneumonic plague cases in the Qinghai-Tibet Plateau, and assuming hypoxia was associated with changes in the pharmacokinetics of streptomycin. After that, we conducted animal experiments to confirm the hypothesis.

## Methods

### Pneumonic plague clinical data from the Tibetan plateau

From January 1, 2000 to December 31, 2012, clinical data of patients with pneumonic plague came from database of the Center for Endemic Disease Control and Prevention of Qinghai Province, was extracted for analysis.

The pneumonic plague diagnostic criteria were as follows:① onset of illness was after close contact with the source of plague infection for a few hours to 2–3 days; ② sudden high fever; ③ symptoms of systemic poisoning, with chest pain, cough, sputum and hemoptysis, dyspnea, and cyanosis; ④ wet lung rales and decreased breath sounds; ⑤ and disproportionate signs and symptoms. The laboratory testing standards were as follows: ① suspected Gram-negative plague bacilli in a sputum smear under the microscope; ②specific nucleic acid determination of plague by PCR; ③ and indirect hemagglutination test for plague antibody (F1) that was positive and elevated four times. All of the confirmed cases, laboratory methods, and results were determined with reference to the health industry standards of the People’s Republic of China [6]

.

### Animals

All experiments were approved by Ethics Committee of People’s Hospital Peking University, and performed in compliance with the Animal Management Rule of the People’s Republic of China, and the Care and Use of the Laboratory Animals Guide of the People’s Hospital Peking University. Twenty-four healthy male SD rats (200 g, Vital River Laboratory Animal Technology Co. Ltd., Peking, China) were used, and they were fed under specific pathogen free circulation. They were randomly divided into hypoxic group and control group, with each group consisting of 12 SD rats. The rats in the hypoxia group were placed in the hypoxic chamber for 30 days, in which the oxygen concentration was maintained at 12.4% ± 0.5%, equivalent to an altitude of 4000 m. The other rats were kept in a normal oxygen chamber for 30 days, in which the oxygen concentration was maintained at 21%.

#### The hypoxia chamber and the normal chamber

In the hypoxia chamber, a solenoid valve controlled the flow of gas (oxygen and high-purity nitrogen) into the chamber [7] .There was an accurate oxygen sensor, a carbon dioxide sensor, and soda lime in the chambers to maintain the oxygen concentration at 12.4% ± 0.5%, the carbon dioxide concentration at 5% ± 0.5%, the temperature at 20–24 °C, and humidity of 40–60%. The normal oxygen chamber was prepared exactly as the hypoxia chamber, except for the oxygen concentration.

#### The normal conditions of animals

All of the rats were weighed before they were put in the chambers and after 30 days. Two milliliters of arterial blood was obtained from the tail artery of each rat and was used for arterial blood gas analysis in a small animal blood gas analyzer (VetStat, Idexx Laboratories, Maine, United States) and for routine blood testing (pocH-100ivd, Sysmex, Japan). Liver and kidney function were also tested using analyzers (VetTest8008, Idexx Laboratories, Maine, United States).

#### Pharmacokinetic study

All of the rats were fasted for 12 h before dosing, and were weighed and injected intraperitoneally by streptomycin 200 mg/kg. Before dosing (0 h) and 20 min, 40 min, 60 min, 80 min, 2 h, 4 h, 6 h, 8 h, 10 h, 12 h post dose, 0.25 mL of blood was collected and preserved in heparinized collection tubes. Then the blood samples were centrifuged at 3000 rpm for 4 min to obtain the plasma, and all plasma samples were stored at − 80 °C until analysis.

### HPLC detection of the plasma streptomycin concentrations

#### Reagents

The reference standards were streptomycin sulfate salt (Product Number: 85884 Sigma Aldrich) and amikacin sulfate (Product Number: A2324 Sigma Aldrich). Sodium 1-hexanosulfonate and sodium phosphate dodecahydrate were obtained from Alfa Aesar (Shore Road, Heysham, Lancs). Acetonitrile (HPLC grade) and 85% phosphoric acid were obtained from DikmaPure (Lake Forest,USA). The water was double-distilled in an all-glass still after passage through an ion-exchange column (Milli-Q).

#### Equipment and HPLC conditions

The chromatographic system consisted of a pump (ESA 582; Molford, MA, USA) equipped with an autosampler (ESA 542 plus) and a SHIMADZU (Kyoto, Japan) UV-VIS detector SPD-20A. The analytical column and the guard column were a Merck RP-18 (particle size 5 μm, 250 mm × 4.6 mm I.D, Merck, KGaA, Germany) and a Merck RP-18E (20 mm × 3 mm I.D), respectively. The column flow-rate was maintained at 1.0 mL/min. The column temperature was set to 40 °C (±0.1). The detector wavelength was 200 ± 0.2 nm. Upon completion of the daily analysis, the column was washed with a mixture of acetonitrile and water (65:35).

The mobile phase for measuring serum streptomycin and amikacin consisted of buffer (20 mM sodium 1-hexanesulfonate and 25 mM tribasic sodium phosphate, pH 3.4, solvent A) and acetonitrile (solvent B) (88:12, *v*/v). The pH solution was adjusted with phosphoric acid (85%) and was filtered through a 0.22 μm filter (Millipore Corp., Bedford, MA) prior to use.

#### Sample preparation

The extraction was performed in plasma from rats, to which amikacin (100 μg/mL) was added as internal standards. The samples were deproteinized by the addition of 20% trichloroacetic acid (TCA, 120 μL/mL of serum). The supernatant was separated from the precipitate by centrifugation at 14000 rpm, 4 °C, for 60 min. A volume of 80 μL of each solution was injected and analyzed for 20 min.

#### Assay validation

The calibration curve for streptomycin (1/x^2^ weighting, linear) ranged from 10 to 500 μg/mL. In assay validations, the intra- and interassay precision (coefficient of variation) values were less than 8%. For streptomycin quality-control (QC) samples, and the intra- and interassay accuracy values were − 9.0 to 8.6%. The recovery was greater than 67.9%. The stability of QC samples under different conditions was evaluated based on peak areas compared with the freshly prepared QC samples, and the results indicate that these analytes were stable.

#### Pharmacokinetic data analysis

The pharmacokinetic study was performed following a single dose of 200 mg/kg streptomycin to twenty-four rats under hypoxic and control conditions. The pharmacokinetic parameters were calculated by noncompartmental analysis using the Phoenix WinNonlin software (version 6.3, Pharsight Corp, Mountain View, CA, USA).

### Statistics

All of the values were presented as means ± SEMs. Differences were defined as statistically significant at *P* < 0.05. Similarly, weight and the results of the blood gas, albumin, creatinine (Cr), blood urea nitrogen (BUN), alanine aminotransferase (ALT), aspartate aminotransferase, erythrocyte counts, and hematocrit analyses were subjected to a paired t-test, and the differences were concluded to be statistically significant at *P* < 0.05. Statistical comparisons were undertaken using Student’s unpaired t-test unless otherwise stated. Significance was defined as P < 0.05.

Statistical analysis for the plasma pharmacokinetic parameters of streptomycin compared the hypoxic condition with the control condition. The primary pharmacokinetic parameters were C_max_ and AUC_0-∞_ on a logarithmic scale. The ratio of least-square (LS) means of the test treatment (hypoxic condition) and reference treatment (control condition) value was calculated, and 90% confidence intervals (CIs) were constructed. Both the ratios of LS means and the 90% CIs were retransformed to the original scale. Treatment effect was considered significant at the 5% level. Hypoxia effect on any pharmacokinetic parameter was considered to exist if 90% CIs of the geometric least-squares mean ratio of the test treatment relative to the reference treatment fell outside the equivalence boundaries of 0.80–1.25.

## Results

### Retrospective analysis of clinical data

From January 1, 2000, to December 31, 2012, there were 32 pneumonic plague patients totally [8, 9] ,which were confirmed by clinical manifestations and sputum culture. Fourteen patients were from Nangqian County in 2004 [10] ,twelve were from Xinghai County in 2009 [11] ,five were from Langxian County in 2010 [11, 12] ,and the last one case was a member of the laboratory staff with improper protective measures in Langxian County. The distribution of incident cases of pneumonic plague showed a population difference without sex differences, as shown in Table 1.

**Table 1:** Distribution of human plague cases and deaths from January 1, 2000 to December 31, 2012 in Qing Hai province, China

Case	From the region	Initial case (Y/N)^a^	Die or not (Y/N)	Normal dose (Y/N)
1	Nangqian	Y	Y(died in 3 days)	N (no streptomycin)
2	Nangqian	Y	Y(died in 3 days)	N (no streptomycin)
3	Nangqian	N	Y(died in 3 days)	N (no streptomycin)
4	Nangqian	N	Y(died in 3 days)	N (no streptomycin)
5	Nangqian	N	Y(died in 3 days)	N (no streptomycin)
6	Nangqian	N	Y(died in 3 days)	N (no streptomycin)
7	Nangqian	N	N	N
8	Nangqian	N	N	N
9	Nangqian	N	N	N
10	Nangqian	N	N	N
11	Nangqian	N	N	N
12	Nangqian	N	N	N
13	Nangqian	N	N	N
14	Nangqian	N	N	N
15	Xinghai	N	Y(died in 5 days)	Y
16	Xinghai	N	Y(died in 5 days)	Y
17	Xinghai	Y	Y(died in 3 days)	N (no streptomycin)
18	Xinghai	N	N	N
19	Xinghai	N	N	N
20	Xinghai	N	N	N
21	Xinghai	N	N	N
22	Xinghai	N	N	N
23	Xinghai	N	N	N
24	Xinghai	N	N	N
25	Xinghai	N	N	N
26	Xinghai	N	N	N
27	Langxian	N	N	N
28	Langxian	N	N	N
29	Langxian	N	N	N
30	Langxian	N	N	N
31	Langxian	N	N	N
32	laboratory staff	N	N	N

^a^Initial case: “Y” means plague symptoms due to direct contact with marmots; “N” means plague symptoms due to closely contact with plague patients

The cause of death in Nangqian was mainly related to the delay of diagnosis. After that, with the increased level of diagnosis in plague, the mortality rate decreased significantly. Only one patient died quickly in Xinghai, and patients with a history of close contact were quickly isolated and treated well, except for 2 patients who were treated with conventional dose of streptomycin

There were 9 patients died from pneumonic plague in these 32 cases, and the all-cause mortality rate was 28.125% (9/32). Two of 9 patients were initial cases, and five of 9 patients were close contacts. These seven people died of delayed diagnosis within three days after onset without treatment of streptomycin (Table 1). The remaining two cases of pneumonic plague survived in the first three days, were diagnosed as plague accurately, then administered normal doses of streptomycin, but died in five days after onset (Tables 1 and 2). We identified no other normal-dose streptomycin medical records for pneumonic plague in the Qinghai-Tibet Plateau.

**Table 2:** General conditions of two patients treated with normal doses of streptomycin

	Case 1	Case 2
Contact history	Yes	Yes
Underlying diseases	No	No
Streptomycin (i.m.)	0.5 g q6h	0.5 g q6h
Symptoms	Fever, cough, chest pain, and hemoptysis	Fever, chills, cough, chest pain, and hemoptysis
Time from diagnosis to death (days)	3	4
Complications	Septic shock	Septic shock
Other treatments	Anti-shock and fluid resuscitation	Anti-shock and fluid resuscitation

The normal doses were as follows: streptomycin: first dose 1 g i.m., 0.5 g q6-8 h i.m.; the large dose of streptomycin: first dose 2 g i.m., then 1 g q6h-q4h i.m. When the body temperature returned to normal, the dose of streptomycin was reduced to 1 g q8h. If the body temperature remained normal for an additional 3 days, the dose of streptomycin was reduced to 1 g q12h. And the combination therapy programs in both groups included ceftriaxone, levofloxacin or moxifloxacin, sulfonamides, tetracycline, and gentamicin [13] .Observation continued for more than three days; when the clinical symptoms were relieved, streptomycin was stopped, or sequential treatment with tetracycline was administered for another three days.

### The normal conditions of the animals

Thirty days later, the mice in the hypoxic group weighed significantly less than the normal control group. Hypoxia could lead to sympathetic activation and decreased appetite. The average weight in the hypoxia group was 357.45 ± 65.003 g, which was significantly lighter than in the control group (431.08 ± 36.806 g, *P* < 0.0001). The red blood cell counts were 9.60 ± 0.563 (10^9^/L) and 8.45 ± 0.743 (10^9^/L) in the hypoxia and control groups, respectively. And hemoglobin levels were 18.22 ± 0.515 (g/L) in the hypoxia group and 16.13 ± 0.289 (g/L) in the control group. Then, hematocrit (HCT) in these groups were 0.57 ± 0.013 and 0.50 ± 0.050 respectively. In addition, there were significant differences between the two groups for red blood cell counts (*P* = 0.007), hemoglobin content (*P* = 0.001) and HCT (*P* = 0.003). However, there were no significant differences in white blood cell (*P* = 0.881) or platelet counts (*P* = 0.404) between the two groups.

Regarding blood gas analysis, the arterial blood pH was not different between the two groups, but there were significant differences in the arterial partial pressure of oxygen, carbon dioxide partial pressure, and sodium bicarbonate ion concentrations between the two groups. The body compensated for the acid-base balance; therefore, the pH was normal, the sodium bicarbonate ion concentration and pCO_2_ decrease, and the pO_2_ increased (Table 3).

**Table 3:** The normal conditions of the animals in the hypoxia and control groups

		Hypoxia	Normal	P value
Weight (g)		357.45 ± 65.003	431.08 ± 36.806	< 0.0001
Complete blood count	RBC (×10^9^/L)	9.60 ± 0.563	8.45 ± 0.743	0.007
	Hb (g/L)	18.22 ± 0.515	16.13 ± 0.289	< 0.0001
	HCT	0.57 ± 0.013	0.50 ± 0.050	0.003
	WBC (×10^9^/L)	11.67 ± 3.376	11.93 ± 2.897	0.881
	PLT (×10^9^/L)	988.71 ± 211.596	823.71 ± 458.479	0.404
Blood gas	pH	7.44 ± 0.036	7.47 ± 0.025	0.356
	PaO_2_ (mmHg)	143.67 ± 16.657	102.67 ± 12.662	0.008
	PaCO_2_ (mmHg)	29.67 ± 3.669	38.33 ± 3.215	0.011
	HCO_3_^−^ (mmol/L)	18.70 ± 2.887	25.53 ± 1.155	0.006

All of the data are expressed as means ± standard deviations; there were significant differences if P < 0.05

Liver function did not change significantly after one large dose of streptomycin. Before bleeding, the ALT in the hypoxia group was slightly higher than in the control group, but the difference was not statistically significant (*P* = 0.338, ANOVA). Intensive bleeding affected the liver function in the hypoxia group. The ALT before bleeding was 47.43 ± 10.309 mmol/L, and it increased to 49.50 ± 8.689 mmol/L. This change was not statistically significant (*P* = 0.682,ANOVA). Streptomycin could combine with albumin; thus, the content of albumin in the blood affected the drug concentration in the bound state. The albumin levels in the hypoxia and control groups were 38.29 ± 1.976 mmol/L and 37.43 ± 2.149 mmol/L, respectively (*P* = 0.452, ANOVA).

Intensive bleeding can impact kidney function and effective circulating blood volume. In the hypoxia group, the Cr and BUN levels were 33.14 ± 7.058 mmol/L and 4.27 ± 0.519 mmol/L before bleeding, and they increased to 41.67 ± 7.394 mmol/L and 5.7 ± 0.636 mmol/L, respectively, after bleeding. These changes were significant, with *P* values of 0.024 and 0.002 (ANOVA), respectively, compared with the control group. There were no differences in BUN levels (*P* = 0.13, ANOVA) or in Cr between the hypoxia group and control group before or after bleeding (*P* = 0.702, ANOVA) (Table 4).

**Table 4:** Effects of bleeding on liver and kidney function

	Hypoxia		Control	
	Before	After	Before	After
ALT (IU/L)	47.43 ± 10.309	49.50 ± 8.689	50.02 ± 8.734	51.38 ± 7.529
Albumin (mmol/L)	38.29 ± 1.976	37.43 ± 2.149	40.67 ± 1.154	38.93 ± 2.005
Cr (mmol/L)	33.14 ± 2.005	41.67 ± 2.005*	34.42 ± 2.005	42.51 ± 6.873*
BUN (mmol/L)	4.272 ± 2.00	5.70 ± .772*	4.892 ± 2.00	5.90 ± 08715*

All data are expressed as means ± standard deviations, *P < 0.05

The 24-h urine samples were collected during bleeding, and there were no statistically significant differences in the urine between the two groups (*P* = 0.456).

### Streptomycin plasma concentrations and pharmacokinetics

Table 5 showed the pharmacokinetic parameters of streptomycin in the hypoxia and control groups. The AUC_0-∞_ in the hypoxia group was 39.513 ch^3^ mg/L/h, which was significantly lower than the control group. After one dose of streptomycin, the absorption rate and extent of hypoxia circulation decreased significantly. The elimination rate constant Ka was an important parameter for the speed of drug elimination in vivo. The K value was 5.078/h in the hypoxia group and 0.129/h in the control group, suggesting that streptomycin was metabolized and eliminated rapidly in hypoxic circumstances. Perhaps hypoxia reduced streptomycin’s efficacy. The peak drug concentration was also significantly different between the two groups; the C_max_ of streptomycin was 177.51 mg/L in the hypoxia group and 310.3 mg/L in the control group.

**Table 5:** Pharmacokinetic parameters of streptomycin in the hypoxia and control groups

Pharmacokinetic parameters	Hypoxia	Control
t1/2	67.618 h	69.828 h
AUC (0-t)	30,562.744 mg/L.h	39,512.611 mg/L.h
AUC (0-∞)	30,562.852 mg/L.h	39,512.611 mg/L.h
Ka	5.078/h	0.129/h
Tmax	20 h	20 h
Vz/F	0.234 L/kg	0.093 L/kg
CLz/F	0 L/h/kg	0 L/h/kg
Cmax	177.51 mg/L	310.3 mg/L

According to the results presented above, the hypoxia group had the following characteristics: decreased AUC and C_max_ and an increased K value. After one dose of streptomycin (200 mg/kg), the drug was metabolized and cleared rapidly, in part because of rapid blood flow. These results might explain why high-dose streptomycin is used to treat pneumonic plague in the Qinghai Plateau.

**Fig. 1:**
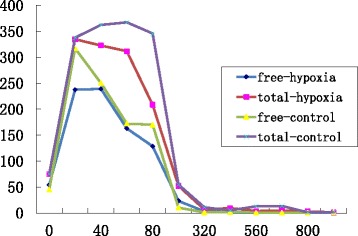
Trends in blood concentrations of streptomycin at different sampling times. *The abscissa represent different time points of blood sampling (units: min), and the ordinate is the drug concentration (units: mg/L). The purple line indicates the total streptomycin concentration in the control group, the red line indicates the total streptomycin concentration in the hypoxia group, the yellow line indicates the free streptomycin concentration in the control group, and the blue line indicates the free streptomycin concentration in the hypoxia group. All the streptomycin concentration data are presented as means ± standard deviations

We examined the total drug and free drug concentrations in rats at different time points of bleeding in both groups, which shown in Fig. 1. The data were shown as means ± standard deviations according to the time points. In Fig. 1, at the 20 min time point after the first bleeding, the concentration of streptomycin reached high levels in the hypoxia and control groups, and the major form of streptomycin was the free drug form in blood. As the experiment continued, the total streptomycin concentration increased in the control group but decreased gradually and plateaued after 80 min in the hypoxia group. The plasma concentration of streptomycin underwent small fluctuations at a lower level after 120 min in both groups, and the streptomycin concentrations were lower in the hypoxia group than in the control group.

## Discussion

Plague is a deadly infectious disease, which causes serious harm to human health. Of all the types of plague, the most serious is pneumonic plague, not only because of its high mortality but also it is one of the greatest hazards in epidemiology [2, 4] .The systemic poisoning symptoms of pneumonic plague include rapid onset, chills, fever, and intense weakness; other clinical manifestations are characterized by respiratory symptoms, including chest pain, cough, hemoptysis, or bloody sputum. In patients with a high degree of cyanosis before death, the skin is dark purple, which is why the disease has been called the Black Death. The Qinghai Plateau is the most active area of plague. The local people are still suffering from plague because the herdsmen often strip and eat marmot; the locals are also poorly informed about plague [14] .Cases of pneumonic plague in the Qinghai Plateau are often severe; they require large doses of streptomycin and combined antibiotics to control the progression of the disease. The WHO recommends streptomycin as the choice of drug used in combination with fluoroquinolones [1, 3] .In the treatment of pneumonic plague, combination therapy is often used to control the disease quickly. Qinghai has a typical highland climate. According to the long-term experience of local doctors and experts in Qinghai plateau, pneumonic plague could not be treated with normal dose streptomycin as in plain area, and they preferred to give high-dose streptomycin to treat these patients. It was not until 2009 that national experts recognized the high-dose of streptomycin program in Xinghai County. New guidelines in China recommended the highest dose of streptomycin for the pneumonic plague is 8 g/d^5^, which is much higher than the 2 g/d recommended by the WHO [1] .Therefore, the efficacy problems of the streptomycin should be studied further in hypoxic environments.

We retrospectively analyzed cases of pneumonic plague in Qinghai since 2000. There were three outbreaks of pneumonic plague in Qinghai Province, which affected a total of 32 patients. Nine of the patients died; thus, the all-cause mortality rate was 28.125%. Epidemics of pneumonic plague were related to close contact with infected marmots.

The largest number of deaths due to pulmonary plague occurred in 2004 in Nangqian County. There were 14 cases; 6 patients died of pneumonic plague, so the all-cause mortality rate in Nangqian was 42.857%. In addition to the 2 initial cases, there were 4 close contacts cases, and all of the 6 cases were undiagnosed. They were treated for normal respiratory infections and only administered penicillin; they also received treatment to reduce their fevers with symptomatic and supportive care. They were not treated effectively and were diagnosed as having pneumonic plague post-mortem. Delayed diagnosis was the main cause of high mortality in Nangqian County. The remaining patients recovered after treatment with high-dose streptomycin.

There were 12 cases of pneumonic plague in 2009 in Xinghai County, and 3 of them died. The initial case died rapidly and soon attracted the attention of the community. Effective measures were taken to isolate and treat these patients. After diagnosis, two of the patients were treated with small doses of streptomycin artificially (first dose: 1 g i.m. and 0.5 g q6h i.m.), and both died of shock and multiple organ failure quickly. These two cases were the only ones received normal dose of streptomycin in the treatment of cases in the Plateau region. Some doctors and health prevention experts in Qinghai had suggested the use of high-dose streptomycin treatment of local plague pneumonia. However, other experts emphasized the side effects of streptomycin, and strongly recommended no high-dose streptomycin. And the only two cases of normal dose streptomycin treatment were followed by the experts from China National Center for Disease Control. After that, they were shocked by the bad outcomes of these two patients.

The subsequent treatments for plague in Langxian County and in the laboratory-staff were smooth and all of the 6 patients survived following the new guideline.

The same medicines showed different therapeutic effects in Plateau and plain areas. Cases of pneumonic plague in the Plateau treated with normal-dose streptomycin could have caused the disease to become uncontrolled, but the cases in the plains could be clinically cured. One case of bubonic plague with secondary pneumonic plague was cured in Inner Mongolia.

From clinical experience in the long-term use of large doses of streptomycin in Qinghai and the two cases of deaths with small doses of streptomycin, we believe that the effective blood concentration of streptomycin in vivo changes due to hypoxia caused by the high altitude.

Previous studies have found that drug metabolism in the body can change under hypoxic conditions. Wolfgang and Ritschel investigated altitude hypoxia effects on the pharmacokinetics of acetazolamide [15] and pethidine [16] at an altitude of 4360 m. The drug clearance (CL) and mean residence time (MRT) of acetazolamide in healthy volunteers were significantly increased in acute and chronic hypoxic conditions compared with volunteers living in the plain areas. In contrast, the apparent volume of distribution (VdD) was significantly reduced. The MRT of pethidine was significantly increased in acute and chronic hypoxia compared to volunteers living in the plains, while CL decreased significantly.

Drug metabolism varies under different hypoxic conditions. Arancibia and colleagues [17] found that the maximum plasma concentration of furosemide was reduced in acute and chronic hypoxia. The AUC, the VdD, and the MRT were reduced under acute hypoxic conditions but increased under chronic hypoxic conditions. However, these parameters were not significantly different, compared with the parameters of the volunteers from the plains. The research of Arancibia et al. on prednisolone contrasted with our study [18, 19] .They studied the pharmacokinetic characteristics of prednisolone under acute and chronic hypoxic conditions in highlands volunteers. They found that the pharmacokinetic characteristics of prednisolone changed significantly, regardless of whether the volunteers first entered the Plateau region at an elevation greater than 3600 m or had been living in the plateau region for six months. The Cmax values were increased by 16.9% and 14.1%, and the AUC increased by 12.8% and 13.5%, while the VdD decreased by 20.4% and 14.6%, and the CL decreased by 25.2% and 15.6%, respectively, in the acute and chronic hypoxic conditions, suggesting that acute and chronic hypoxia could affect the pharmacokinetics of prednisolone in the human body.

In previous studies of the effects on drug metabolism of hypoxic circumstances in animals and humans, different drugs have performed differently in acute or chronic hypoxia. In our research, long-term hypoxic animals were administered a single dose of streptomycin. The absorption, utilization, and effective blood concentration of streptomycin were lower than under normal oxygen conditions. Drug metabolism was more rapid under hypoxic conditions, its effective utilization was reduced (AUC was significantly smaller), and the clearance rate increased by 50 times (K value in the hypoxia group was significantly higher than in the control group), indicating that streptomycin was cleared rapidly and was less utilized in vivo. In addition, the Cmax was only half of the concentration in normoxic animals, and the effective peak blood concentration was reduced. Therefore, the doses of streptomycin must have been increased to achieve the same efficacy. In addition, the excretion of streptomycin from the kidneys increased in long-term hypoxic animals (there were differences, but not statistically significant). Thus, in the Plateau regions, the doses of streptomycin might need to be larger to maintain effective plasma concentrations, which could also explain why there were no obvious side effects in plague patients or in various tissues and organs after the use of large doses of streptomycin in the Plateau regions because the drug concentration was low. Therefore, in the Qinghai Plateau, the effective drug concentration of streptomycin might have been affected by the impact of reduced oxygen concentrations, and only large doses of streptomycin could achieve effective treatment.

In addition, as an aminoglycoside antibiotic, streptomycin is excreted unchanged and only part is bound to albumin; the remainder is in the free drug form, which is distributed rapidly by the blood circulation to different tissues and organs and is then discharged through the urine. In long periods of hypoxia, to maintain a normal oxygen supply, the velocity of the body and the respiratory rate and depth were accelerated; therefore, drugs were excreted rapidly.

## Conclusions

Our results confirm that high doses of streptomycin are required for the treatment of pneumonic plague in the Qinghai Plateau, which is related to the altered metabolism of streptomycin in hypoxic environments. Our research has several limitations that must be improved. Only two cases with normal dose are a very small number of patients to be analyzed. However, we can’t carry out clinical trials for normal dose of streptomycin on more plague patients, because plague is a terrible disease in these areas. Although there were only two patients treated with normal dose of streptomycin in Qinghai Plateau, we learned a painful lesson from them. Since then, the maximum dosage of streptomycin in the Chinese plague guideline has been modified.

We chose adult SD rats that were raised under normoxic conditions before the experiment and then were kept under hypoxic conditions for 30 days, which was equivalent to a long-term hypoxia period for residents of the plains live that lived on the Plateau for longer than 3 months. This situation was very different from that of the people who live in the highlands permanently.

Our experimental conditions and experimental design were subject to many aspects, and many variables were considered: the resistance of *Y. pestis* to streptomycin, the difference between continuous medication and a single dose, and the extent to which binding between streptomycin and albumin is affected by hypoxia.

Animal experiments cannot adequately reflect the situation in the human body. More experiments will be required, such as animal experiments performed on animals from the plateau regions and human pharmacokinetic experiments in the plains and plateau regions.

## References

[1] Plague manual-epidemiology, distribution, surveillance and control. Wkly Epidemiol Rec 1999;74:447. 10635759

[2] Butler T (2009). Plague into the 21st century. Clin Infect Dis.

[3] Bossi P, Tegnell A, Baka A (2004). Bichat guidelines for the clinical management of plague and bioterrorism-related plague. Euro Surveill.

[4] Gao ZC (2011). Current diagnostic and therapeutic status of human plague [in Chinese]. Zhonghua Jie He He Hu Xi Za Zhi.

[5] Ministry of Health Emergency Response Office and Chinese Center for Disease Control. Emergency manual plague prevention and control. Peking University Medical Press. 2009:221.

[6] Health Department of the People's Republic of China. Plague Diagnostic Criteria. Health Industry Standard of the People’s Republic of China. WS279–2008.

[7] Chen L, Cao ZL, Han F (2010). Chronic intermittent hypoxia from pedo-stage decreases glucose transporter 4 expression in adipose tissue and causes insulin resistance. Chin Med J.

[8] Wang H, Wang G, Wang Z (2004). Retrospection and present state of plague prevention and control in Qinghai Province about 50 years. Chin. J Epidemiol..

[9] Wei RJ (2011). Characteristics of patients infected with plague in Qinghai and prevention. Chin J Prev Med.

[10] Wang GJ, Li C, Wang H (2007). Lung plague: an analysis on 14 cases in Qinghai Province in 2004. Chin J Epidemiol.

[11] Wu KM, Yang YH, Wang YZ (2011). Epidemiological analysis of plague in Qinghai province between 2000 and 2009. Chin J Epidemiol.

[12] Dawa W, Pan WJ, Gu XY (2011). clinical features, diagnosis and treatment of 5 cases of primary pneumonic plague in Tibet in 2010 [in Chinese]. Zhonghua Jie He He Hu Xi Za Zhi.

[13] Zhang G, Zhang GJ, Liu ZC (2006). Status and Progress of antibiotic treatment of plague. Chin J Epidemiol.

[14] Li M, Wang GJ, Cui BZ (2006). Plague of Qinghai and its epidemic analyse from 1996 to 2005. Chin J Epidemiol.

[15] Ritschel WA, Paulos C, Arancibia A (1998). Urinary excretion of acetazolamide in healthy volunteers after short- and long-term exposure to high altitude. Methods Find Exp Clin Pharmacol.

[16] Ritschel WA, Paulos C, Arancibia A (1996). Pharmacokinetics of meperidine in healthy volunteers after short- and long-term exposure to high altitude. J Clin Pharmacol.

[17] Arancibia A, Nella Gai M, Paulos C (2004). Effects of high altitude exposure on the pharmacokinetics of furosemide in healthy volunteers. Int J Clin Pharmacol Ther.

[18] Arancibia A, Gai MN, Chávez J (2005). Pharmacokinetics of prednisolone in man during acute and chronic exposure to high altitude. Int J Clin Pharmacol Ther.

[19] Gai MN, Pinilla E, Paulos C (2005). Determination of prednisolone and prednisone in plasma, whole blood, urine, and bound-to-plasma proteins by high-performance liquid chromatography. J Chromatogr Sci.

